# 2017 ESC guidelines for the management of acute myocardial infarction in patients presenting with ST-segment elevation: comments from the Dutch ACS working group

**DOI:** 10.1007/s12471-018-1134-0

**Published:** 2018-07-04

**Authors:** F. Arslan, L. Bongartz, J. M. ten Berg, J. W. Jukema, Y. Appelman, A. H. Liem, R. J. de Winter, A. W. J. van ’t Hof, P. Damman

**Affiliations:** 1 Department of Cardiology, St. Antonius Hospital, Nieuwegein, The Netherlands; 2 Department of Cardiology, Haaglanden Medical Center, Den Haag, The Netherlands; 3 Department of Cardiology, Leiden University Medical Center, Leiden, The Netherlands; 4 Department of Cardiology, VU Medical Center, Amsterdam, The Netherlands; 5 Department of Cardiology, Franciscus Gasthuis, Rotterdam, The Netherlands; 6 Department of Cardiology, Academic Medical Center, Amsterdam, The Netherlands; 7 Department of Cardiology, Maastricht University Medical Center, Maastricht, The Netherlands; 8 Department of Cardiology, Zuyderland Medical Center, Heerlen, The Netherlands; 9 Department of Cardiology, Radboud University Medical Center, Nijmegen, The Netherlands

**Keywords:** STEMI guidelines, NVVC ACS working group statement

## Abstract

On behalf of the Dutch ACS working group, we discuss the most important changes in recommendations in the 2017 ESC guidelines for the management of acute myocardial infarction in patients presenting with ST-segment elevation relevant for both the general and interventional cardiologist.

## Introduction

The 2017 ESC guidelines for the management of acute myocardial infarction in patients presenting with ST-segment elevation (STEMI) were presented at the 2017 European Society of Cardiology (ESC) Conference in Barcelona and published in the *European Heart Journal* [1]. Compared with the 2012 version, the new guidelines incorporate significant changes in recommendations. In this article, we focus on the changes most relevant for daily practice.

## Thrombus aspiration

The 2012 guideline based its ‘should be considered’ recommendation for routine thrombus aspiration on small trials and single-centre studies. The current guideline does not recommend routine manual thrombus aspiration, based on the TOTAL [2] and TASTE [3] trials. These two studies are the largest (10,064 and 7,244 patients respectively) and most recent to investigate both the efficacy and safety of manual thrombus aspiration.

In the TOTAL trial, a randomised trial of routine aspiration thrombectomy with percutaneous coronary intervention (PCI) versus PCI alone in patients with STEMI undergoing primary PCI, a high (70%) retrieval of macroscopic thrombus was achieved and a large proportion (nearly 80%) of the patients included had a high thrombus burden (TIMI (thrombolysis in myocardial infarction) thrombus grade 4 or 5). However, the trial did not show any effect on cardiovascular death, recurrent MI, cardiogenic shock, or class IV heart failure during 1 year follow-up. In addition, a significantly higher rate of stroke, mainly within 180 days after intervention, was observed in patients undergoing manual thrombus aspiration (1.2%).

The TASTE (thrombus aspiration during STEMI) trial showed no difference in all-cause mortality, cardiac death, rehospitalisation for MI or stent thrombosis 1 year after randomisation, even in subgroups with a high thrombotic burden; approximately 30% of the study population was classified as having TIMI thrombus grade 4 or 5. In a recent meta-analysis incorporating these trials, the lack of benefit for thrombus aspiration was confirmed [4]. In this meta-analysis, a benefit of thrombus aspiration with regard to death in patients with a high thrombus burden could not be ruled out.

Given these concordant results in two high-powered, large, randomised trials and confirmed by a large meta-analysis, the ESC guideline correctly discourages the use of routine manual thrombus aspiration. In cases of high thrombus burden, the use of a thrombus aspiration device is left to the discretion of the interventional cardiologist according to the new guideline.

## Multivessel coronary revascularisation

The 2017 guideline gives a class IIA recommendation (‘should be considered’) for complete revascularisation in patients presenting with STEMI and multivessel disease, which is approximately 50% of the STEMI population [5, 6]. Treatment of the infarct-related artery (IRA) improves clinical outcomes, but evidence for improved prognosis with immediate revascularisation (preventive PCI) of remaining lesions is lacking. The new guideline summarises recent trials investigating complete revascularisation either during the index procedure [7, 8] or staged during hospitalisation [9, 10]. All but the Preventive Angioplasty in Acute Myocardial Infarction (PRAMI) trial show no improvement in clinical outcomes such as mortality or re-infarction, but a consistent and significant reduction in repeat revascularisation with preventive PCI. In the PRAMI trial, patients undergoing primary PCI were randomised to preventive PCI or no preventive PCI. Subsequent PCI for angina was recommended only for refractory angina with objective evidence of ischaemia. A reduction was observed with preventive PCI with regard to non-fatal MI, refractory angina and the composite end-point, including cardiac death. However, the group with incomplete revascularisation included more diabetics with anterior wall MIs, both important determinants of clinical outcomes after acute MI. Recent meta-analyses confirm the lack of benefit for clinical outcomes [11, 12].

The end-point repeat revascularisation of non-IRAs is determined by the treatment strategy itself and may not reflect the clinical benefit of preventive PCI over no PCI. Even with objective ischaemia such as evidenced by fractional flow reserve (FFR), deferral of a significant lesion that likely causes symptoms should not count as an end-point, since the occurrence of the event (revascularisation) is related to the lesion and not to the treatment arm (preventive PCI vs no PCI).

Complete revascularisation in STEMI patients with cardiogenic shock is an area of great interest with little but conflicting data in non-randomised studies [13, 14]. The recent Culprit Lesion Only PCI versus Multivessel PCI in Cardiogenic Shock (CULPRIT-SHOCK) trial is the first randomised study to assess culprit-only versus multivessel PCI in 706 patients presenting with STEMI and cardiogenic shock. The rate of composite death or renal-replacement therapy was lower among patients who underwent culprit-only PCI than in those who underwent multivessel PCI. The better outcome was mainly driven by lower mortality in the culprit-only PCI group [15].

Given the consistent results of recent trials and meta-analyses, the Dutch ACS (acute coronary syndromes) working group endorses the ESC recommendation by adding that patient factors such as complaints, haemodynamic stability, renal function, lesion complexity as well as local facilities, logistics (working hours with more back-up available) and economic considerations should also be taken into account in the decision-making process. We believe that an ischaemia-driven staged revascularisation (either during hospitalisation or elective) is an effective alternative.

## Early P2Y_12_ inhibitor administration

As stated in the ESC guidelines, the evidence for pre-loading P2Y_12_ inhibitors in STEMI patients is not very substantial.

One small randomised trial (337 patients) was conducted comparing the efficacy of a high loading dose of 600 mg clopidogrel given in the pre-hospital phase versus administration after coronary angiography. The results showed a trend towards better outcomes with regard to death, re-infarction or urgent target vessel revascularisation in favour of pre-treatment, but no difference in vessel patency. In addition, no difference in bleeding complications was observed [16].

The ATLANTIC (Administration of Ticagrelor in the Cath Lab or in the Ambulance for New ST-Elevation Myocardial Infarction to Open the Coronary Artery) trial investigated whether pre-treatment with ticagrelor versus treatment in the catheterisation laboratory was associated with better outcomes [17]. Patients with ongoing STEMI of less than 6 h duration were randomised to pre-hospital (in the ambulance) versus in-hospital (in the catheterisation laboratory) treatment with ticagrelor. There was no difference in the primary end-point of resolution of ST-segment elevation or improved TIMI flow pre-PCI; however ST resolution after PCI (pre-specified secondary outcome) was significantly improved [18]. The study was underpowered for clinical end-points, including mortality. However, definite stent thrombosis at 30 days was significantly less frequent in pre-treated patients (0.2% vs 1.2%; *p* = 0.026). This difference was already apparent in the first few days after PCI. The ATLANTIC trial shows that pre-hospital ticagrelor administration a short time before PCI in patients with ongoing STEMI is safe but does not improve pre-PCI coronary reperfusion. It may, however, improve ST resolution after PCI and reduce the risk of post-PCI stent thrombosis.

The following should be noted when interpreting the ATLANTIC trial. The mean difference in time of administration in the ATLANTIC trial between the two groups was only 31 min. With regard to the pharmacokinetic data, ticagrelor and prasugrel are deemed effective after 30 min up to 4 h after administration. Furthermore, Valgimigli et al. [18] showed that inhibition of platelet aggregation by prasugrel in STEMI patients is suboptimal for at least 2 h after administration. This is the primary reason why pre-treatment during transport is standard practice in many European countries. Of note, a small study by Parodi et al.. [19] showed better platelet inhibition after ingestion of crushed ticagrelor tablets than with whole tablets.

Based on the limited available data, the current ESC guidelines state that the earliest possible administration may be preferable to achieve early efficacy. In the Netherlands, where pre-hospital diagnosis of STEMI has a high accuracy owing to the digital transfer of electrocardiograms from the ambulance for consultation, the Netherlands Society of Cardiology (NVVC) ACS working group supports this statement.

When there is doubt about the STEMI diagnosis, or there is a high probability that cardiac surgery will be necessary (for example in cases where a mechanical complication is suspected), withholding the loading dose should be considered by the cardiologist until a more definitive diagnosis is made.

## Intravenous ß‑blockers prior to primary PCI

The ESC guidelines give a class IIa, level of evidence A recommendation for intravenous beta-blockade at the time of presentation in patients without contra-indications, no signs of acute heart failure, and with a systolic blood pressure of more than 120 mm Hg.

In the Effect of Metoprolol in Cardioprotection During an Acute Myocardial Infarction (METOCARD-CNIC) trial, 270 patients with anterior STEMI without signs of overt heart failure and a systolic blood pressure of more than 120 mm Hg were randomised to receive either 15 mg of intravenous metoprolol or nothing at time of diagnosis [20]. The primary end-point was infarct size assessed by MRI at 5–7 days. MRI was performed in 88% of patients. Infarct size was significantly smaller in patients treated with intravenous metoprolol, but the standard deviation was large: 25.6 ± 15.3 g after intravenous metoprolol versus 32.0 ± 22.2 g in controls; (*p* = 0.012). This resulted in a higher ejection fraction at 6 months (48.7% vs 45.0%; *p* = 0.018), and a significant decrease in patients with an ejection fraction ≤35% and a class I indication for an implantable cardioverter-defibrillator. Limitations of this study are that only anterior STEMI was included, and that it was not blinded or placebo-controlled.

The Early Intravenous Beta-Blockers in Patients with ST-Segment Elevation Myocardial Infarction Before Primary Percutaneous Coronary Intervention (EARLY-BAMI) trial tried to confirm these findings by including all types of STEMI patients in a randomised, placebo-controlled trial [21]. The trial randomised 683 patients with STEMI within 12 h of onset to intravenous metoprolol (5 mg at recruitment and an additional 5 mg immediately before PCI) or placebo. No significant decrease was found in infarct size measured by creatinine kinase release or by MRI, the primary end-point. MRI data was, however, only available in 66% of patients, mainly due to logistical reasons. Early intravenous metoprolol was associated with a borderline reduction of malignant ventricular arrhythmias (3.6% vs 6.9%; *p* = 0.050).

Reasons for the lack of effect on infarct size might be that, compared to the METOCARD-CNIC trial, infarct size was smaller overall and that around 19% of patients were already using a beta-blocker before inclusion. Furthermore, the beta-blocker dose in the METOCARD-CNIC trial was higher and administered in the ambulance. A subanalysis of the METOCARD-CNIC trial suggests that the timing of beta-blocker administration plays a role in determining its effect on infarct size, with the greatest effect seen in patients receiving beta-blockade earlier before reperfusion [22]. However, with the strict inclusion and exclusion criteria used, intravenous beta-blockade did not cause an increase in adverse events.

The ESC guidelines give a class IIa, level of evidence A recommendation for intravenous beta-blockers. Summarising the above results, the use of intravenous beta-blockers before primary PCI seems safe in patients without hypotension, heart failure or cardiogenic shock. However, no robust data is available with regard to a benefit of this treatment, with one study showing a reduction in infarct size. We believe there is currently no indication for routine early intravenous beta-blockers in STEMI, even in the absence of the above-mentioned contra-indications.

## Specific interventional cardiology themes

### Radial access

In the 2017 guidelines, the radial artery is now considered the preferred access site with a class I recommendation (level of evidence A). The latest MATRIX (Minimising Adverse Haemorrhagic Events by TRansradial Access Site and Systemic Implementation of angioX) trial not only showed reduced bleeding complications, but also confirmed a significant survival benefit over transfemoral access as observed in earlier studies such as the RIVAL (RadIal Vs femorAL access for coronary intervention) and RIFLE-STEACS (Radial Versus Femoral Randomized Investigation in ST-Elevation Acute Coronary Syndrome) trials. The NVVC ACS working group endorses the recommendation for the radial approach as the default access site in ACS patients undergoing primary PCI by operators with experience using this approach.

### DES over BMS

The 2017 guidelines now also give a class I recommendation for the use of drug-eluting stents (DES) over bare metal stents (BMS), given the lower risk of re-infarction and target vessel revascularisation with DES. The latest generation of DES have also been shown to have a lower risk of stent thrombosis and recurrent MI compared to the first-generation DES.

### Time delays and limits

The 2017 guidelines have replaced the term ‘door-to-balloon’ time by ‘first medical contact (FMC) to wire crossing’ as a clinical performance measure. FMC is defined as the initial assessment of the patient by a physician, paramedic, nurse or other trained emergency medical system personnel who can obtain and interpret the electrocardiogram, and perform the initial intervention (e. g. defibrillation). The target for diagnosing STEMI is set at <10 min after FMC. For the primary PCI strategy, the 2017 guidelines dictate a < 90 min target from STEMI diagnosis to wire crossing. The time limits for primary PCI after symptom onset have also changed slightly. The 2017 guidelines give a class I (level of evidence A) recommendation for reperfusion therapy in all patients with symptoms of ischaemia of <12 h duration and persistent ST-segment elevation. A class IIa recommendation (‘should be considered’) is given for routine primary PCI for patients presenting late (12–48 h) after symptom onset. In asymptomatic patients, routine PCI of an occluded IRA > 48 h after onset of STEMI is not indicated (class III, level of evidence A).
